# Cell line-specific features of 3D chromatin organization in hepatocellular carcinoma

**DOI:** 10.5808/gi.23015

**Published:** 2023-06-30

**Authors:** Yeonwoo Kim, Hyeokjun Yang, Daeyoup Lee

**Affiliations:** Department of Biological Sciences, Korea Advanced Institute of Science and Technology, Daejeon 34141, Korea

**Keywords:** chromatin loops, compartments, hepatocellular carcinoma, *in situ* Hi-C, TADs, 3D chromatin organization

## Abstract

Liver cancer, particularly hepatocellular carcinoma (HCC), poses a significant global threat to human lives. To advance the development of innovative diagnostic and treatment approaches, it is essential to examine the hidden features of HCC, particularly its 3D genome architecture, which is not well understood. In this study, we investigated the 3D genome organization of four HCC cell lines—Hep3B, Huh1, Huh7, and SNU449—using *in situ* Hi-C and assay for transposase-accessible chromatin sequencing. Our findings revealed that HCC cell lines had more long-range interactions, both intra- and interchromosomal, compared to human mammary epithelial cells (HMECs). Unexpectedly, HCC cell lines displayed cell line-specific compartmental modifications at the megabase (Mb) scale, which could potentially be leveraged in determining HCC subtypes. At the sub-Mb scale, we observed decreases in intra-TAD (topologically associated domain) interactions and chromatin loops in HCC cell lines compared to HMECs. Lastly, we discovered a correlation between gene expression and the 3D chromatin architecture of *SLC8A1*, which encodes a sodium-calcium antiporter whose modulation is known to induce apoptosis by comparison between HCC cell lines and HMECs. Our findings suggest that HCC cell lines have a distinct 3D genome organization that is different from those of normal and other cancer cells based on the analysis of compartments, TADs, and chromatin loops. Overall, we take this as evidence that genome organization plays a crucial role in cancer phenotype determination. Further exploration of epigenetics in HCC will help us to better understand specific gene regulation mechanisms and uncover novel targets for cancer treatment.

## Introduction

In 2022, the American Cancer Society estimated that approximately 800,000 people were diagnosed with liver cancer and 700,000 died from the disease worldwide. Liver cancer is the third leading cause of cancer death, partly reflecting that the lack of accurate liver cancer-specific diagnostic tools has limited early diagnosis [1,2]. There has recently been increasing interest in immunotherapy and targeted therapy [3], but the diagnosis and treatment of liver cancer remain challenging. Developing novel diagnostic tests and treatments is critical for improving the prognosis of patients. Therefore, we must better understand the genomic characteristics of liver cancer.

The most common type of primary liver cancer is hepatocellular carcinoma (HCC), which is a malignant tumor of hepatocytes. The causes of HCC are very diverse; they include chronic infection with hepatitis B virus or hepatitis C virus, alcohol abuse, and metabolic disease [4]. This heterogeneity makes standardized chemotherapy ineffective, resulting in frequent metastases to nearby organs, poor prognoses, and high mortality rates [5]. Researchers have thoroughly scrutinized and categorized the transcriptomes of numerous HCC tissue samples [6-8], but we still know relatively little about the relevant three-dimensional (3D) interactions between distant gene loci, which have been shown to play a crucial role in regulating gene expression [9].

The mammalian genome maintains a highly organized and dynamic 3D form that arises from two kinds of interactions [10]. At the megabase (Mb) scale, the genome is partitioned into A and B compartments. Active chromatin, which possesses high-transcription histone markers and many genes, is called the A compartment, whereas inactive chromatin, which is gene-poor and possesses gene-silencing histone markers, is called the B compartment [11]. At the sub-Mb level, each compartment is divided into smaller, self-interacting domains called TADs (topologically associated domains), which are insulated from neighboring domains [12-14]. Although the precise function of TADs is not yet fully understood, it is believed that they contribute to regulating gene expression by helping ensure that cis-regulatory elements and their target promoters are brought into proximity with one another [15]. Therefore, from the perspective of epigenetics, studying 3D chromatin organization is essential to understanding the mechanisms of gene expression regulation.

To characterize the 3D chromatin landscape of HCC cell lines, we performed *in situ* Hi-C and assay for transposase-accessible chromatin sequencing (ATAC-seq) of four HCC cell lines and compared the results to those of a normal epithelial cell line. This comprehensive analysis of the chromatin interactions in HCC extends our knowledge of genome topology and epigenetics in hepatocellular carcinoma.

## Methods

### Cell culture

This research used four HCC cell lines: Hep3B, Huh1, Huh7, and SNU449 (Table 1). The HCC cell lines were cultured and harvested in the laboratory of Kyung Hyun Yoo at Sookmyung Women’s University. We used human mammary epithelial cells (HMECs) from normal female mammary tissue as non-cancer control samples (primary mammary epithelial cells; normal, human; ATCC PCS-600-010). HMECs were cultured in mammary epithelial cell basal medium (ATCC PCS-600-030) supplemented with components from a mammary epithelial cell growth kit (ATCC PCS-600-040). All cell lines were grown at 37°C in a humidified incubator with 5% CO_2_.

### Cell harvest

We harvested cells with 0.05% trypsin-EDTA, resuspended 5 million cells in 5 mL 1× phosphate buffered saline (PBS), added 274 μL of 36.5% formaldehyde (final concentration, 2%), mixed the solution thoroughly by inversion, and incubated the suspended cells at room temperature for 10 min. We added 400 μL of 2.5 M glycine and placed the sample on ice for 15 min. Cells were pelleted by centrifugation at 500 ×g for 5 min, the supernatant was discarded, and the cells were resuspended in 5 mL 1× PBS. The cells were equally distributed to five new tubes (1 million cells/tube) and pelleted by centrifugation, and the supernatant was discarded.

### *In situ* Hi-C and library sequencing

We followed the Arima-HiC protocol (cat No. A160259, Arima Genomics, Inc., San Diego, CA, USA) to perform *in situ* Hi-C with 1 million harvested cells. We generated a library using an Arima-HiC kit (cat No. A510008, Arima Genomics, Inc.) and sequenced the library using the 150 bp paired-end method of the Illumina NovaSeq 6000 system (Illumina, San Diego, CA, USA).

### Assay for transposase-accessible chromatin sequencing

Harvested nuclei of 50,000 cells were incubated in 25 μL fresh TD buffer (10 mM Tris-HCl, pH 8.0, 5 mM MgCl_2_, and 10% dimethylformamide) with 2.5 μL Tn5 transposase for 30 min at 37°C. We purified DNA fragments with a QIAquick PCR purification kit (cat No. 28106, Qiagen, Hilden, Germany) and amplified the library using a KAPA HiFi HotStart ReadyMix (KK2061, Roche, Mannheim, Germany) as described in the provided manual, with adjustment of the PCR cycle number. The resulting libraries were purified with a QIAquick PCR purification kit. The purified libraries of HCC cell lines were sequenced via the 150 bp paired-end method of the Illumina NovaSeq 6000 system. The 150 bp paired-end method of the Illumina HiSeq 2500 system was used for HMECs.

### Data processing and analysis

#### *In situ* Hi-C analysis

The *in situ* Hi-C sequencing data were analyzed using HiC-Pro [16]. Each raw file was aligned to the human genome (hg19) and filtered. Replicate data were merged. Contact matrices were built from the merged data, and iterative correction and eigenvector decomposition (ICE) normalization were applied. Various resolutions (10, 20, 40, 100, and 500 kb) of ICE-normalized Hi-C matrices were generated, and annotation files indicating genomic bins were developed. Contact probability calculation and principal component analysis (PCA) to define the compartment were done using Cooltools at a 100 kb resolution [17]. To define TAD boundaries, reciprocal insulation (RI) scores were calculated using CaTCH at 20 kb resolution [18]. For most of the Hi-C analyses, including relative contact probability analysis, TAD insulation scoring, aggregate TAD analysis, and aggregate peak analysis, GENOVA was used [19]. To identify chromatin loop interactions, the HiCCUPS algorithm of juicer tools was used with default parameters [20].

#### ATAC-seq analysis

For ATAC-seq analysis, the adaptors of raw reads were trimmed with Cutadapt [21], and the trimmed sequences were mapped to the human genome (hg19) via Bowtie2 (version 2.5.0) with default parameters [22]. The aligned bam files were merged and sorted with SAMtools [23]. The bam2wig tool of the RSeQC tool was used to generate bigwig files [24].

#### Total RNA-sequencing analysis

For the total RNA-sequencing (RNA-seq) analysis, raw reads were aligned to the human genome (hg19) using STAR (version 2.7.10) with default parameters [25]. Cufflinks (fr-firststrand) was used to analyze differential expression levels [26]. CummeRbund was used to create certain plots [27]. Additional plots, including box plots, were drawn with the R package, ggplot2 [28]. Heatmaps were drawn with Java Treeview [29], and the examples of genome-wide data were visualized using the Integrative Genomics Viewer (IGV) [30].

### Public data acquisition

Publicly released total RNA-seq data were downloaded from NCBI Gene Expression Omnibus (GEO) datasets. These files were obtained in fastq format. The total RNA-seq data of Hep3B, Huh1, Huh7, and SNU449 cells (GSM2551564, GSM2551568, GSM2551570, and GSM2551589, respectively) were obtained from GSE97098 [31]. The total RNA-seq data of HMECs (GSM5667415) were obtained from GSE187119 [32].

### Data availability

The Hi-C and ATAC-seq datasets have been deposited in the NCBI GEO; http://www.ncbi.nnlm.nih.gov/geo/) under accession number GSE226217 (SuperSeries). This SuperSeries (GSE226217) is composed of two SubSeries: GSE226215 (ATAC-seq) and GSE226216 (Hi-C).

## Results

### Long-range interactions are increased in HCC cell lines

**Fig. 1.**
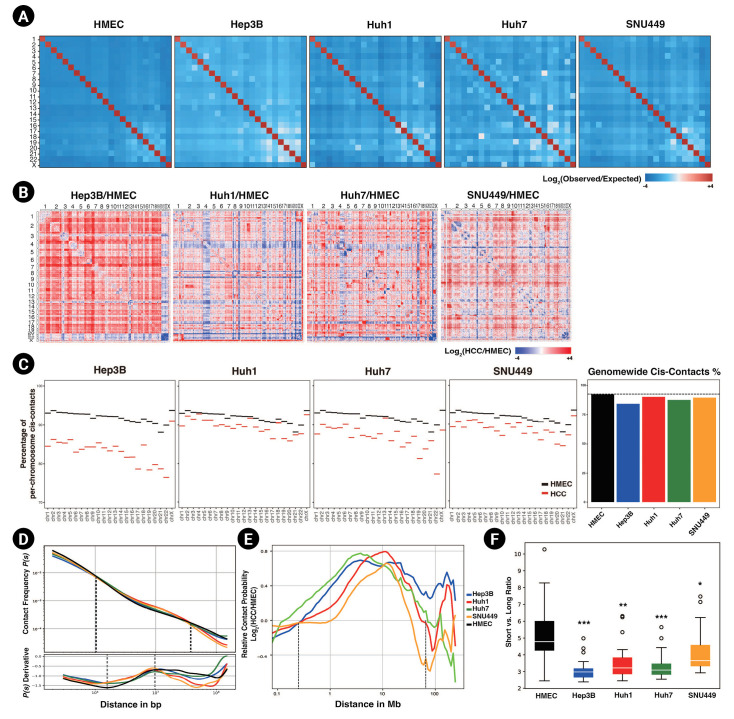
Long-range interactions are increased in hepatocellular carcinoma (HCC) cell lines. (A) Genome-wide Hi-C contact maps representing trans-contacts between chromosomes at a 500 kb resolution, normalized by expected contact counts. (B) Contact maps depicting a log2-fold change in contacts of HCC cell lines compared to those of human mammary epithelial cells (HMECs). (C) The percentages of cis-contacts per chromosome in HCC cell lines and HMECs are shown (left). A bar plot of averaged cis-contact ratios across the whole genome, with the ratio of cis-contacts in HMECs presented as a dashed line (right). (D) Averaged contact probabilities according to the genomic distance are shown, with dashed lines representing points at which the contact probabilities of HMECs and HCC cell lines are the same (top). The derivatives of the contact probability are shown in the subpanel, and the regions of minimum and maximum points are marked with dashed lines (bottom). (E) Relative contact probability (plots represent a log2-fold change in contact probability between HMECs and HCC cell lines according to the genomic distance. The dashed lines mark the points with no fold change. (F) A box plot of the ratios between short-range cis contacts (shorter than or equal to 1 Mb) and long-range cis contacts in HCC cell lines and HMECs. p-values were calculated using the Wilcoxon rank sum test (^*^p < 0.005, ^**^p < 1.0e-4, ^***^p < 1.0e-5).

To investigate the 3D chromatin organization of HCC cell lines, we performed *in situ* Hi-C in four HCC cell lines and HMECs (Fig. 1), with two biological replicates for each cell line. We compared genome-wide Hi-C features between HCC cell lines and HMECs. We found significantly more long-range interactions, including intrachromosomal and interchromosomal interactions, in all four HCC cell lines relative to HMECs. Long-range interactions especially increased in Hep3B and Huh7, compared to Huh1 and SNU449.

First, the trans-interactions (i.e., interchromosomal interactions) were explored. Our results revealed that HCC cell lines had higher observed versus expected (obs/exp) trans-contact ratios (Fig. 1A) than HMECs. All trans-contact counts were much lower than expected in the case of HMECs, but not in HCC cell lines. The whole-contact maps, which compared the ratio of interactions throughout the genome between HCC cell lines and HMECs, also reflected this difference (Fig. 1B). Most trans-bins indicated higher contact frequencies in HCC cell lines. Along with these differences in the contact maps, we observed that all chromosomes of HCC cell lines had decreased cis-contact percentages compared to HMECs (Fig. 1C). In other words, in HCC cell lines, the number and ratio of trans-contacts in most chromosomes were consistently higher than those of HMECs. Previous studies reported that cancer genomes undergo various chromosomal rearrangements, including chromosome duplications, deletions, and translocations [33,34]. To prevent chromosomal rearrangement from affecting our result, we calculated copy number variations (CNVs) using HiCnv [35] (Supplementary Table 1, Supplementary Fig. 1). Consistent with our previous results, HCC cell lines had higher trans-contacts in contact maps with no CNV regions (Supplementary Fig. 1A).

Markedly, the contact probabilities of HCC cell lines and HMECs changed as the genomic distance passed 1 Mb (Fig. 1D and 1E). When the genomic distance was shorter than or equal to 1 Mb, the contact probability was higher for HMECs than for HCC cell lines. As the genomic distance reached and then passed 1 Mb, the contact probability of HMEC shrank until the distance reached 40 Mb. Based on this observation, we divided intrachromosomal contacts into short- and long-range contacts based on a genomic distance of 1 Mb. The ratios between short vs. long-range interactions of HCC cell lines were significantly lower than those of HMECs, indicating that HCC cell lines had dominant long-range interactions (Fig. 1F, Supplementary Fig. 1D). In conjunction with frequent interchromosomal contact, HCC cell lines also revealed intensified contacts in long-range intrachromosomal interactions.

From the derivative of the contact probability plot, we could further infer the size of TADs and the linear density of cohesin [36]. The average TAD sizes, determined from the maximum points in the derivative plots, were slightly increased in HCC cell lines (Fig. 1D subpanel). The cohesin linear density, which was determined by the depth of the minimum point, was decreased in HCC cell lines; this could be interpreted as indicating the presence of weaker intra-TAD interactions based on the previous study [36], which found stronger TADs and chromatin loops were associated with more robust insulation and a higher density of cohesins. In summary, in HCC cell lines, shorter contacts (e.g., intra-TAD interactions) were decreased, and more elongated contacts (e.g., interchromosomal contacts) were increased. This change might cause the abnormal cancer phenotype of HCC cell lines.

### Distinguishing compartment landscapes in HCC cell lines

Having observed a discrepancy in genome-wide chromosome contact between HMECs and HCC cell lines, we next defined compartmental domains to explore potential relationships between contact differences and 3D chromatin organization. We also analyzed open chromatin regions via ATAC-seq using two technical replicates.

**Fig. 2.**
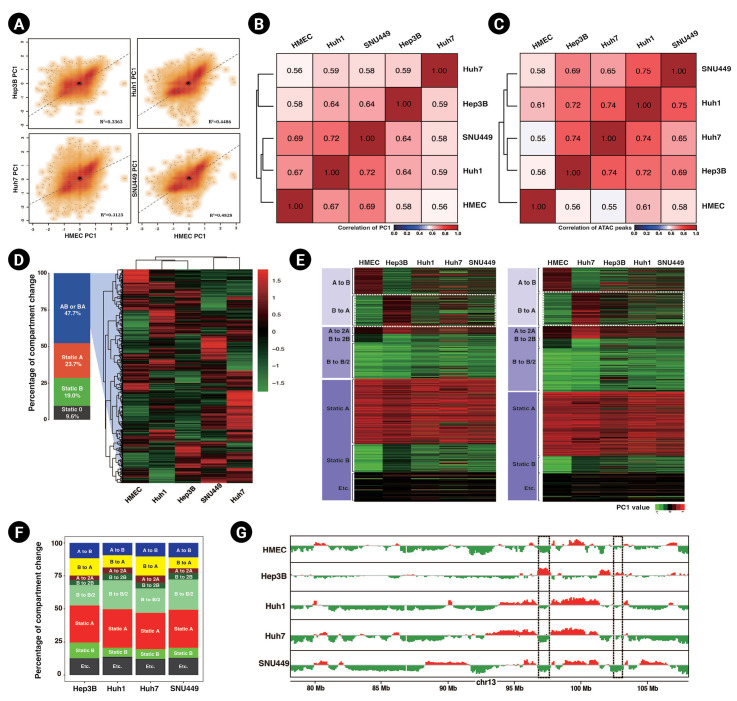
Distinguishing compartment landscapes in hepatocellular carcinoma (HCC) cell lines. (A) Scatter plots of the compartment scores (PC1) of HCC cell lines relative to human mammary epithelial cells (HMECs) at a 100 kb resolution. Linear regression lines and correlation coefficients (R^2^) are presented. (B) A heatmap showing Pearson correlations of PC1 correlation coefficients with hierarchical clustering. (C) A Pearson correlation heatmap generated from ATAC peaks with hierarchical clustering. (D) An accumulative column graph of the ratio of compartmental changes between HCC cell lines and HMECs (left). The genomic bins of CCs are depicted as a heatmap with hierarchical clustering (right). (E) Heatmaps of compartment scores in each cell type, sorted according to compartment alteration. The genomic bins were sorted by compartmental changes between HMECs and Hep3B cells (left) and between HMECs and Huh7 cells (right). (F) Accumulative column graphs of the percentages of different compartment transitions from HMECs to each HCC cell line. (G) Example of compartmental domains of HMECs and HCC cell lines. The example domains of cell line-specific CCs are marked with a dotted box.

We analyzed compartments through PCA analysis of Hi-C contacts at a 100 kb resolution. Positive PC values were taken as defining A compartments, and negative values were taken as delimiting B compartments [37]. The PC1 values of HCC cell lines showed moderate positive correlations with those of HMECs (Fig. 2A). Based on these scores, we calculated the Pearson correlations for hierarchical clustering (Fig. 2B). Huh1 and SNU449 cells had the strongest correlation with one another. Moreover, Huh1 and SNU449 cells had the lowest correlation scores with Huh7. To compare these results to the correlation scores obtained from the ATAC peaks, we mapped the trimmed ATAC-seq fastq files onto the hg19 genome and merged the mapping results of the replicates. After sorting and indexing, we calculated the Pearson correlation scores at a 100 kb resolution. The ATAC-seq peaks showed close correlations between SNU449 and Huh1 cells, followed by Huh7 and Hep3B cells, and thus supported the PCA analysis results (Fig. 2C).

Regarding compartmentalization changes between HMECs and HCC cell lines, we found that almost 50% of the compartments in HMECs were altered (A-to-B or B-to-A; called changed compartments or CCs) in HCC cell lines (Fig. 2D). Intriguingly, the CCs were unique in each HCC cell line. For example, some B-to-A domains in Hep3B were not changed in the other HCC cell lines. Accordingly, we analyzed the CCs in each cell line. Surprisingly, each cell line had only 25% CCs compared to HMECs, compared to the 50% difference between all HCC cell lines and HMECs (Fig. 2F). Thus, the CCs appeared to be distinctive to each HCC cell line.

Next, we generated heatmaps that sorted bins according to the comparison between HMECs and each HCC cell line (Fig. 2E, Supplementary Fig. 2). The A-to-B domains of Hep3B cells were mostly consistent with those of the other HCC cell lines, whereas the B-to-A domains of Hep3B cells only marginally overlapped with those of other HCC cell lines. For instance, in chromosome 13, there was a B-to-A domain in Hep3B cells that corresponded to a static B domain in the other HCC cell lines (Fig. 2G). Additionally, the PC values of most compartment B domains were lower in HCC cell lines than in HMECs. Unlike the compartment scores for compartment B, those for compartment A did not decrease by more than half in HCC cell lines compared to HMECs (Fig. 2E).

To summarize, compartment alterations in HCC cell lines were mostly found in the B compartment of HMECs, which were weakened or changed to A compartment regions in the HCC cell lines. Notably, these B-to-A domain changes were cell line-specific. Our results suggest that compartment analysis could potentially be used to classify the subtype of HCC, which is critical for selecting a cancer treatment strategy.

### Intra-TAD interactions are decreased in HCC cell lines

**Fig. 3.**
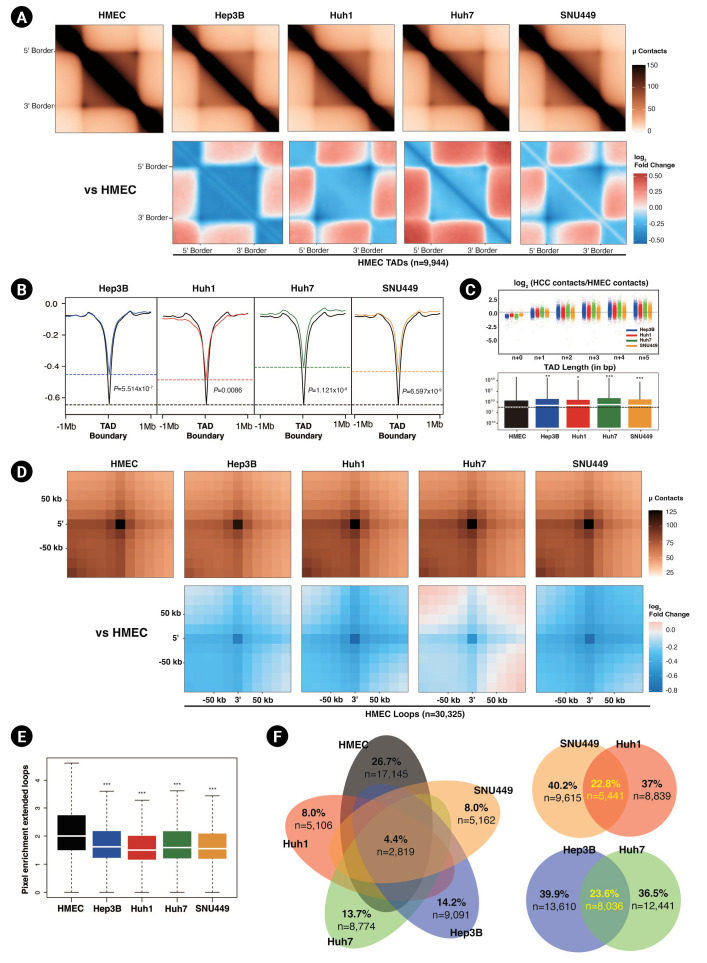
Diminished intra-TAD (topologically associated domains) interactions in hepatocellular carcinoma (HCC) cell lines. (A) Aggregate TAD analysis (ATA) of the normalized contacts (top) and differential interactions (bottom) of HCC cell lines compared to human mammary epithelial cells (HMECs) in 9,944 HMEC TADs. (B) Averaged insulation scores at TAD boundaries of HMEC within ±1 Mb. Dashed lines mark the insulation scores of HMECs (black) and HCC cell lines at HMEC TAD boundaries. Calculated p-values with the Wilcoxon rank sum test are shown. (C) TAD n+1 plots (top) showing inter-TAD interactions with neighbor TADs of HCC cell lines compared to HMECs. The dashed line is located at zero. The box plot shows the TAD length distribution for each cell line (bottom). The medians of the TAD lengths are represented with white lines, and the median for HMECs is shown with a black dashed line. p-values were calculated using the Wilcoxon rank sum test (^*^p < 0.05, ^**^p < 0.001, ^***^p < 1.0e-6). (D) Aggregate peak analysis of the normalized peaks (top) and differential peaks (bottom) of HCC cell lines compared to HMECs at 30,325 HMEC peaks within ±50 kb. (E) A box plot showing the distribution of pixel enrichments in ATA plots, with median values represented by white lines. p-values were calculated using the Wilcoxon rank sum test (^***^p < 2.2e-16). (F) Venn diagrams represent the similarity of peaks between HMECs and HCC cell lines.

Next, we explored shorter-range neighborhood interactions, namely TADs and chromatin loop interactions, in greater depth. Based on our initial results, we expected smaller TAD sizes and weaker intra-TAD interactions in HCC cell lines compared to HMECs. To analyze this in more detail, we defined TADs by RI analysis using CaTCH [18]. HMECs had more abundant (n = 9,944) TADs than HCC cell lines (Hep3B = 8,473, Huh1 = 9,159, Huh7 = 7,693, SNU449 = 9,553). As we expected, according to ATA analysis, the intra-TAD interactions were diminished in HCC cell lines compared to HMECs (Fig. 3A). Subsequently, we calculated the insulation score, which provided an aggregate of interactions within a sliding square across the interval [18]. The insulation scores' local minima were considered to be the TAD boundaries. The insulation scores for TAD interactions of HCC cell lines were also higher (less negative), i.e., had smaller signal amplitude than those of HMECs, implying that the insulating abilities at TAD borders were affected under HCC (Fig. 3B). Huh7 and SNU449 cells exhibited particularly smaller signal amplitude at TAD boundaries than Huh1 and Hep3B cells. Weaker TAD borders could explain why the inter-TAD interactions and TAD sizes were increased in HCC cell lines compared to HMECs (Fig. 3C). Hep3B and Huh7 cells had greater inter-TAD strength and longer average TAD lengths than Huh1 and SNU449 cells.

Another critical interaction is the chromatin loop interaction. Chromatin loops are formed by the interaction between the pairs of loci that show significantly higher contact frequencies than their neighbors [11]. We defined loops via the HiCCUPS algorithm of juicer tools [20]. We detected 30,325 chromatin loops in HMECs and 21,646, 20,477, 14,280, and 15,056 chromatin loops in Hep3B, Huh7, Huh1, and SNU449 cells. Similar to the weaker TAD interactions, we observed that HCC cell lines had fewer and weaker chromatin loops than HMECs (Fig. 3D and 3E). The intensity and number of chromatin loops in SNU449 and Huh1 cells were significantly lower than in Hep3B and Huh7 cells.

To prevent chromosomal rearrangement from affecting our result, we also analyzed TAD domains and chromatin loops after excluding CNVs from the genome (Supplementary Table 1, Supplementary Fig. 1B–1F). The results were consistent with our previous results about the characteristics of TAD and chromatin loops of HCC cell lines compared to HMECs.

We also examined the similarities between chromatin loop interactions. Unexpectedly, only 4.4% of loop interactions were common to all four HCC cell lines and HMECs (Fig. 3F). Hep3B and Huh7 cells had the highest number of common peaks, followed by Huh1 and SNU449 cells. When we combined the results of all Hi-C analyses, including those of genome-wide contacts, compartments, ATAC-seq peaks, and TAD characteristics, the HCC cell lines clearly segregated into two subgroups based on chromatin 3D organization: one group comprising SNU449 and Huh1 cells and one comprising Hep3B and Huh7 cells. This tendency was also observed in the previous study, which identified six HCC subgroups through unsupervised transcriptome analysis [38].

### Alteration of 3D chromatin organization can disturb gene expression

**Fig. 4.**
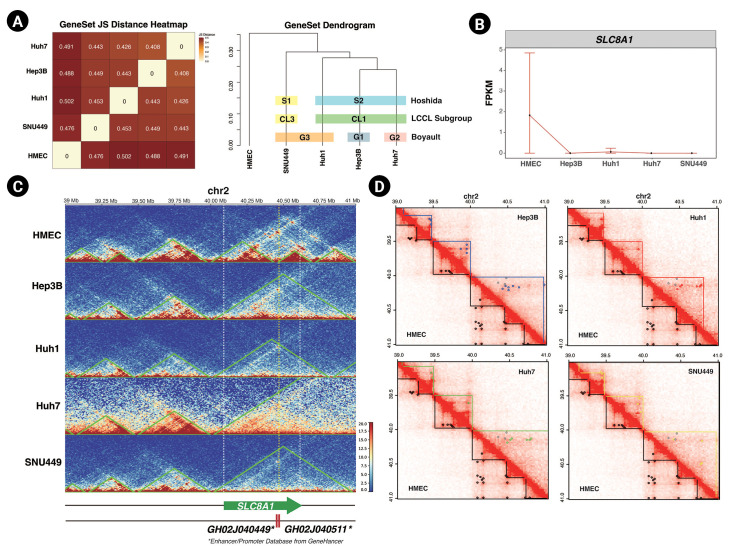
Changes in 3D chromatin organization can disturb gene expression. (A) A JS Distance correlation plot (left) and a dendrogram (right) of differentially expressed genes genes in hepatocellular carcinoma (HCC) cell lines and human mammary epithelial cells (HMECs). Three previous HCC subtyping strategies are indicated by colored boxes. (B) FPKM (fragments per kilobase of transcript per million) gene-level plot of *SLC8A1* in HMECs and HCC cell lines. (C) Normalized Hi-C contact maps with TADs (topologically associated domains; green lines) indicated, spanning 34 to 41 Mb of chromosome 2 in the five cell lines. The white dashed lines mark the *SLC8A1* locus, and the yellow dashed line marks the location of enhancers. (D) Normalized Hi-C contact maps indicating TADs (straight lines) and chromatin loops (dots) of HCC cell lines and HMECs. The TAD boundaries of each cell line and chromatin loops are marked with the following colors: HMEC, black; Hep3B, blue; Huh1, red; Huh7, green; and SNU449, yellow. The gray dots in the upper triangle mark the HMECs’ chromatin loops.

Finally, we assessed the relationship between 3D chromatin organization and gene expression. We used the public total RNA-seq results for HMECs, Hep3B, Huh1, Huh7, and SNU449 cells and mapped them to the hg19 genome using STAR [25]. We applied Cufflinks to identify differentially expressed genes (DEGs) of five cell lines compared to each other [39] and used cummeRbund to plot the results of our analysis [27]. We calculated the correlation of DEGs in HCC cell lines and HMECs (Fig. 4A left panel). The DEGs of Hep3B and Huh7 cells had the strongest relationship with each other, while those of HMECs had the weakest relationship with the DEGs of the HCC cell lines. This result was also depicted as a dendrogram (Fig. 4A right panel). There are three published strategies for subtyping HCC based on transcriptome analysis, namely the subgroupings reported by Boyault et al. [38] (G1 to G6, G-standard), Hoshida et al. [40] (S1 to S3, S-standard), and Caruso et al. [41] (CL1 to CL3, CL-standard). Based on G-standard, Huh1 and SNU449 are G3, Hep3B is G1, and Huh7 is G2. Based on S-standard, Hep3B, Huh1, and Huh7 are S2 and SNU449 is S2. Finally, based on CL-standard, Hep3B, Huh1, and Huh7 are CL1 and SNU449 is CL3. When taking together all these previous studies, SNU449 has the least similarity with other HCC cell lines, and Hep3B and Huh7 tend to be the most similar in the transcriptome. In our RNA-seq analysis, Hep3B and Huh7 had the highest correlation, and SNU449 had the lowest correlation with other HCC cell lines. Since our RNA-seq analysis results were closely related to three previous subtypings, we could conclude that this public RNA-seq data and our analysis were sufficient to support the observation of transcription level change. Importantly, as described earlier, Hep3B and Huh7 cells demonstrated the highest correlation in 3D chromatin organization and RNA-seq analysis, followed by Huh1 and SNU449 cells. This implies that 3D chromatin organization could also be another HCC subtyping standard.

Our RNA-seq analysis revealed that Hi-C analysis results could potentially be used as subtyping standards and supported the connection between 3D chromatin organization and gene expression level. Additionally, we found the expression level of *SLC8A1* (encoding Solute Carrier Family 8 A1, chr2: 40,097,270-40,611,053) was lower in all tested HCC cell lines compared to HMECs (Fig. 4B). Notably, the TAD domain at this locus was altered in HCC cell lines relative to HMECs (Fig. 4C). In HMECs, the boundaries of the TAD containing *SLC8A1* were located at 40 Mb and 40.46 Mb of chromosome 2. The two most highly ranked enhancers for *SLC8A1* are GH02J040449 and GH02J040511, located at 40,449,400 and 40,511,348 bp, respectively, and are thus found in the same TAD as *SLC8A1* in HMECs. In HCC cell lines, however, the TAD of *SLC8A1* was enlarged. We speculate that this altered TAD might not be able to support proximity between the enhancers and the promoter of *SLC8A1* and that the smaller TAD might be necessary for the proper expression of *SLC8A1*.

Moreover, the chromatin loops annotated in the *SLC8A1* TAD of HMECs were not detected in the HCC cell lines. These discrepancies in 3D chromatin organization might form a basis for the differential expression of *SLC8A1* in HCC cell lines. Furthermore, we found another gene, *CHDR1* (encoding Cadherin related family member 1, chr10:84,194,635–84,219,621), that expression level reduction and TAD disruption both occurred (Supplementary Fig. 3). The low expression of *CHDR1* is an unfavorable prognostic factor, and overexpression of *CHDR1* could inhibit glioma cell growth [42]. In Hep3B, Huh1, and Huh7, the TAD at the *CHDR1* gene locus enlarged. In SNU449, *CHDR1* TAD was separated into two smaller TADs. Moreover, all HCC cell lines lost chromatin loop interaction at the *CHDR1* locus. These disruptions of TADs and chromatin loops might cause the suppression of gene expression. These examples support the idea that there is a relationship between gene expression and 3D chromatin organization and further emphasize the importance of characterizing 3D chromatin organization in cancer.

## Discussion

The study of epigenetics in HCC is essential for several reasons. Firstly, as epigenetic changes play a critical role in the development and progression of HCC [43], the study of such changes could critically help us understand the mechanisms underlying this disease. Secondly, targeting specific epigenetic changes could be a strategy for improving the diagnosis and prognosis of HCC [44]. Finally, knowledge of HCC epigenetics could facilitate the development of new therapies and personalized medicine, such as epidrugs, by guiding researchers in leveraging relevant DNA methylation inhibitors, histone deacetylases, and other chromatin-modifying enzymes [45]. Therefore, the study of epigenetics in HCC has the potential to provide new insights into the nature of HCC and reduce the burden of this devastating disease worldwide.

*In situ* Hi-C allowed us to characterize the 3D genome organization of four HCC cell lines (Hep3B, Huh1, Huh7, and SNU449) compared to HMECs. The HCC cell lines were found to have the following changes relative to HMECs: a higher frequency of long-range (>1 Mb) interactions, such as trans-interactions and intrachromosomal interactions; cell line-specific compartmental changes relative to HMECs; and reductions in the number and strength of TADs and chromatin loops. Using the correlation scores for all 3D chromatin structures, we could divide the four HCC cell lines into two subgroups: one comprising Hep3B and Huh7 cells and one comprising Huh1 and SNU449 cells. This subtyping was supported by the results of our RNA-seq analysis. Finally, we revealed that the gene expression level and 3D chromatin organization are linked in the case of *SLC8A1*, which encodes a sodium-calcium antiporter that plays a crucial role in inducing apoptosis by increasing the influx of calcium ions [46-48].

We previously studied 3D chromatin organization in different breast cancer cells and tissues [49]. Both breast cancer and HCC cell lines showed increases in trans-contacts and distant interactions relative to those of HMECs’. The TAD insulation scores and peak strength at chromatin loops were decreased in cancer cells compared to HMECs. Reduced local contacts and increased global contacts may be common features of cancer cells. However, there were some differences between the two cancer types. In the breast cancer study, the BT549 cell line, which is a triple-negative breast cancer cell line, showed the most distinctive CCs from other breast cancer cells. In the present study, in contrast, the HCC cell lines all had similar A-to-B compartmental changes but cell line-specific B-to-A changes. This distinct B-to-A compartmental change may be a biomarker of HCC cell lines and could potentially be used to classify HCC cell lines.

Although we found some apparent cell line-specific 3D chromatin organizations in HCC, our study has limitations. We used HMECs as a control; as these cells originated from mammary tissue, our findings may not be cancer-specific features but rather liver-specific. Furthermore, we do not report on other epigenetic data, such as histone ChIP-seq results; thus, the results of our Hi-C analysis must be confirmed by additional experiments. Nevertheless, we herein reported distinguishing features of chromatin organization between HCC cell lines and showed that their contact characteristics coincided with those of the previously studied breast cancer cell lines. Finally, the utilized Hi-C analysis pipeline was one that had been previously verified by many studies; thus, our findings should be meaningful in a general sense.

Our results provide new epigenetic perspectives into HCC pathology and cancer biology that can be further explored through future research. There is a long-standing debate over whether cancer cell lines demonstrate phenotypes identical to cancer cells obtained from tissues [50], and studies have shown that a given cell line can display varying characteristics depending on the culture environment [51]. As a result, researchers are making significant efforts to build *in vitro* microenvironments for cancer cells, such as with 3D culture techniques [52]. As an extension of our research, by comparing the monolayer-cultured cancer cell lines, 3D-cultured cancer cell lines, and cancer cells from patients’ tissue samples, we will be able to distinguish three distinct epigenetic phenotypes. Moreover, we will identify the universal features of cancer cells compared to normal cells and determine which epigenetic markers can be used to discriminate cancer cell lines from tissues and normal cells. These findings can serve as a foundation for further research in cancer epigenetics and epigenetic drug development.

## Figures and Tables

**Table 1.** Characteristics of cell lines

Cell line	Host	Morphology	HBV integration
Hep3B	Black male, 8 y	Epithelial	Positive
Huh1	Japanese male, 53 y	Epithelial-like	Positive
Huh7	Japanese male, 57 y	Epithelial-like	Negative
SNU449	Korean male, 52 y	Epithelial	Positive
HMEC	Adult female breast tissue	Epithelial	Negative

HBV, hepatitis B virus.

## Supplementary Materials

Supplementary Table 1. Copy number variations (CNVs) for cell lines

Supplementary Fig. 1. Long-range interactions are increased, and intra-TAD (topologically associated domains) interactions are diminished in hepatocellular carcinoma (HCC) cell lines.

Supplementary Fig. 2. Distinguishing compartment landscapes in hepatocellular carcinoma cell lines.

Supplementary Fig. 3. Changes in 3D chromatin organization can disturb gene expression.
