# Supplementary material for: Cell line-specific features of 3D chromatin organization in hepatocellular carcinoma
Source: Genomics Inform. 2023 Jun 30;21(2):e19. doi: 10.5808/gi.23015 (PMC10326539; doi:10.5808/gi.23015)
Supplement: Supplementary Table 1. — Copy number variations (CNVs) for cell lines [file gi-23015-Supplementary-Table-1.pdf]

Supplementary Table. 1. Copy number variations (CNVs) for cell lines

Cell line	Chromosome	Start	End	Segment value	CNV type
HMEC	chr6	28375090	33244145	-0.499114024	Deletion
HMEC	chr6	161955366	170905599	-0.499114024	Deletion
Huh1	chr4	7248568	18738376	-0.459516383	Deletion
Huh1	chr4	52896002	60147045	-0.459516383	Deletion
Huh1	chr4	88659216	88796193	-0.459516383	Deletion
Huh1	chr4	167235725	168951450	-0.459516383	Deletion
Huh1	chr6	28375090	33244145	-0.639516383	Deletion
Huh1	chr6	110731915	113408682	-0.639516383	Deletion
Huh1	chr8	154794	38736262	-0.521596029	Deletion
Huh1	chr9	0	31620105	-0.629516383	Deletion
Huh1	chr12	85248285	97385863	0.490483617	Amplification
Huh1	chr14	49927882	62182707	0.650483617	Amplification
Huh1	chr18	58446619	78077248	-0.619516383	Deletion
Huh1	chr21	9429449	15084736	-0.689516383	Deletion
Huh1	chr21	40555666	39998203	-0.509516383	Deletion
Huh1	chr21	44477327	48129895	-0.509516383	Deletion
Huh1	chrX	0	28471271	-0.669516383	Deletion
Huh1	chrX	71860000	84235556	-0.469516383	Deletion
Huh1	chrX	90053688	95426672	-0.62709214	Deletion
Huh1	chrX	125505668	143313266	-0.49790675	Deletion
Huh1	chrX	154827135	155225619	-0.669516383	Deletion
Huh7	chr2	141740946	173285793	0.803271298	Amplification
Huh7	chr4	31764916	45133197	-0.536728702	Deletion
Huh7	chr4	182078555	191154276	-0.536728702	Deletion
Huh7	chr6	28375090	33244145	-0.646728702	Deletion
Huh7	chr6	164598868	170905599	-0.646728702	Deletion
Huh7	chr7	43930	28889991	0.543271298	Amplification
Huh7	chr7	38646877	48739733	0.543271298	Amplification
Huh7	chr8	154794	23286630	-0.516024283	Deletion
Huh7	chr8	50580788	56615242	-0.456728702	Deletion
Huh7	chr9	22644515	30584068	-0.456728702	Deletion
Huh7	chr16	0	8499130	-0.456728702	Deletion
Huh7	chr16	48799571	33486529	-0.456728702	Deletion
Huh7	chr18	19230068	24940677	-0.476728702	Deletion

Huh7	chr18	28545526	35615130	-0.529062036	Deletion
Huh7	chr18	40257194	53045088	-0.606728702	Deletion
Huh7	chr18	62332004	78077248	-0.57682579	Deletion
Huh7	chr21	9429449	9877199	-0.616728702	Deletion
Huh7	chr21	46712351	48129895	-0.616728702	Deletion
Huh7	chr22	16096563	22505074	2.486509393	Amplification
Huh7	chr22	39664975	48575500	0.673271298	Amplification
Huh7	chrX	0	155225619	-0.596728702	Deletion
SNU449	chr1	187867524	193376070	0.577889908	Amplification
SNU449	chr1	192984065	244525079	0.577889908	Amplification
SNU449	chr4	160265401	191154276	-0.512110092	Deletion
SNU449	chr6	28375090	32272046	-0.642110092	Deletion
SNU449	chr6	92786930	94599030	-0.642110092	Deletion
SNU449	chr6	170252653	170905599	-0.642110092	Deletion
SNU449	chr9	14769001	25565787	-0.662110092	Deletion
SNU449	chr9	140246881	141100719	-0.662110092	Deletion
SNU449	chr10	20680752	47623336	0.817889908	Amplification
SNU449	chr16	0	634438	-0.542110092	Deletion
SNU449	chr17	44781659	70833591	0.6535683	Amplification
SNU449	chr18	77171751	78077248	-0.452110092	Deletion
SNU449	chr22	48575500	49295484	-0.492110092	Deletion
SNU449	chrX	0	8293118	-0.57147718	Deletion
SNU449	chrX	57900864	72241125	-0.462110092	Deletion
SNU449	chrX	89313384	143280491	-0.509831546	Deletion
Hep3B	chr3	30669088	97010795	-0.478294889	Deletion
Hep3B	chr7	8755336	57927499	0.741705111	Amplification
Hep3B	chr13	19191186	19839527	-0.528294889	Deletion
Hep3B	chr13	45740349	76570847	-0.528294889	Deletion
Hep3B	chrX	0	155225619	-0.669136503	Deletion

HMEC, human mammary epithelial cell.

The location and the type of labeled CNVs in each cell line are shown. All of these regions were excluded in Supplementary Fig. 1.
