# Supplementary material for: Cell line-specific features of 3D chromatin organization in hepatocellular carcinoma
Source: Genomics Inform. 2023 Jun 30;21(2):e19. doi: 10.5808/gi.23015 (PMC10326539; doi:10.5808/gi.23015)
Supplement: Supplementary Fig. 1. — Long-range interactions are increased, and intra-TAD (topologically associated domains) interactions are diminished in hepatocellular carcinoma (HCC) cell lines. [file gi-23015-Supplementary-Figure-1.pdf]

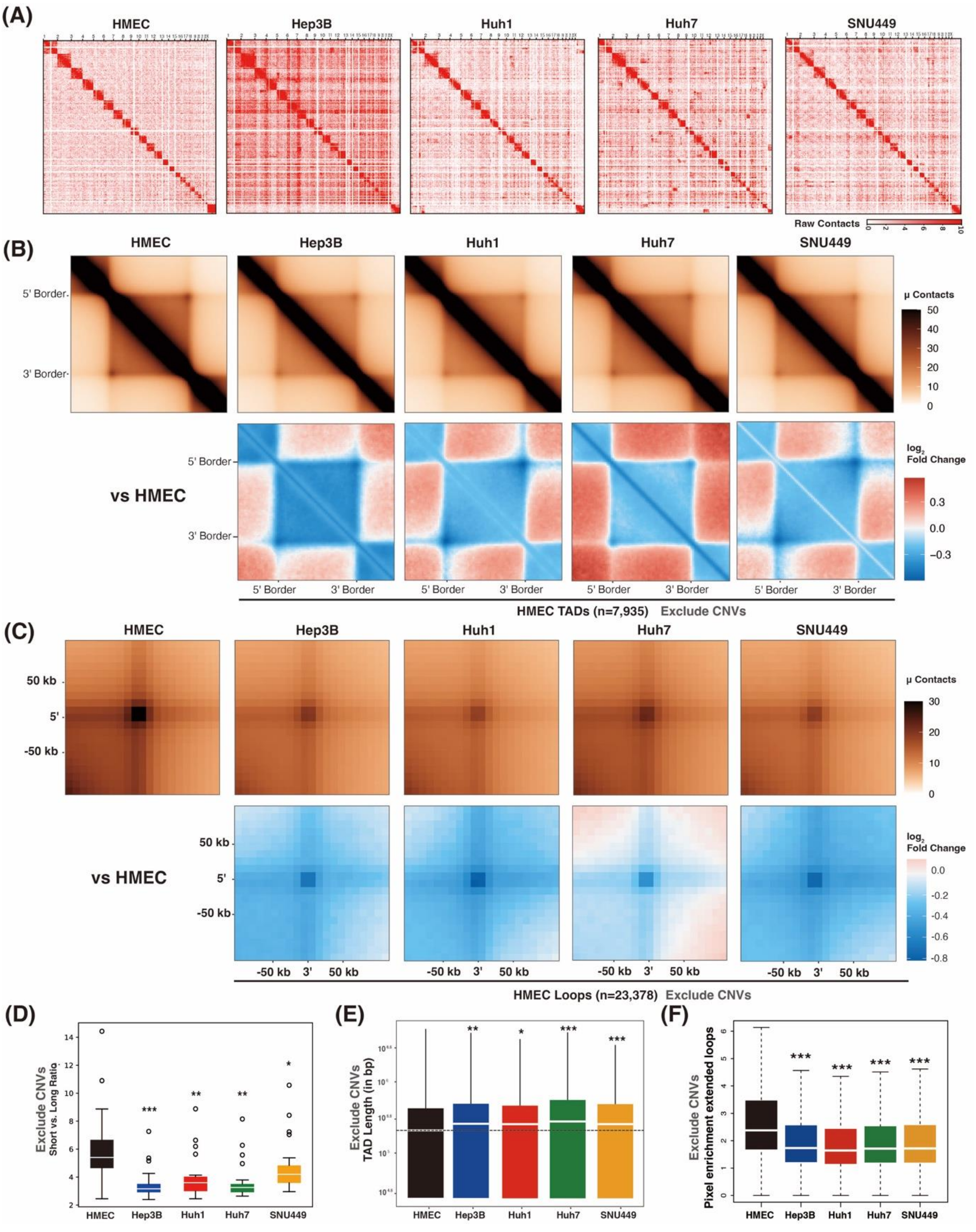

Supplementary Fig. 1. Long-range interactions are increased, and intra-TAD (topologically associated domains) interactions are diminished in hepatocellular carcinoma (HCC) cell lines. (A) Genome-wide Hi-C raw contact maps at a 500 kb resolution. (B) Aggregate TAD analysis (ATA) showing the normalized interactions (top) and differential interactions (bottom) at Copy number variation (CNV)-excluded 7,935 human mammary epithelial cell (HMEC) TADs. (C) Aggregate peak analysis (APA)

showing the normalized interactions (top) and differential interactions (bottom, right) at CNV-excluded 23,378 HMEC chromatin loops. (D) A box plot of the ratios between short-range cis contacts (shorter than or equal to 1 Mb) and long-range cis contacts in HCC cell lines compared to HMECs after masking CNV regions. p-values were calculated using the Wilcoxon rank sum test (*$p < 0.005$, **$p < 10^{-4}$, ***$p < 10^{-5}$). (E) The box plot shows the TAD length distribution after excluding CNVs. The medians of the TAD lengths are represented with white lines, and the median of HMEC is shown with a black dashed line. *P*-values were calculated using the Wilcoxon rank sum test (*$p < 0.05$, **$p < 10^{-3}$, ***$p < 10^{-7}$). (F) A box plot displaying the average normalized contact quantified at the center region within ±1 bin of CNV-excluded 23,378 HMEC chromatin loops.
