# Supplementary material for: Cell line-specific features of 3D chromatin organization in hepatocellular carcinoma
Source: Genomics Inform. 2023 Jun 30;21(2):e19. doi: 10.5808/gi.23015 (PMC10326539; doi:10.5808/gi.23015)
Supplement: Supplementary Fig. 2. — Distinguishing compartment landscapes in hepatocellular carcinoma cell lines. [file gi-23015-Supplementary-Figure-2.pdf]

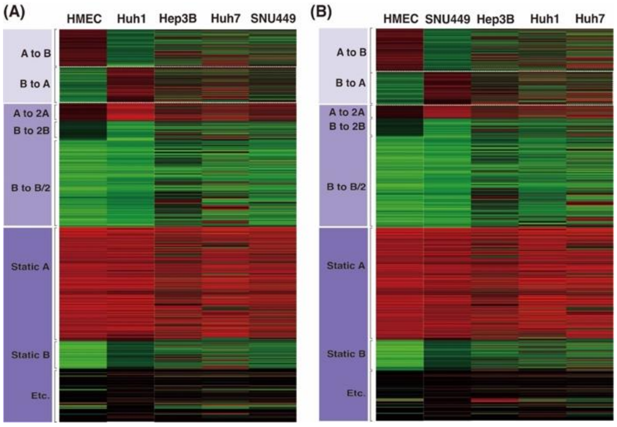

Supplementary Fig. 2. Distinguishing compartment landscapes in hepatocellular carcinoma cell lines. Heatmaps of compartment scores in each cell type, sorted according to compartment alteration. (A) The genomic bins were sorted by compartmental changes between human mammary epithelial cells (HMECs) and Huh1 cells. (B) The genomic bins were sorted by compartmental changes between HMECs and SNU449 cells.
