# Supplementary material for: Cell line-specific features of 3D chromatin organization in hepatocellular carcinoma
Source: Genomics Inform. 2023 Jun 30;21(2):e19. doi: 10.5808/gi.23015 (PMC10326539; doi:10.5808/gi.23015)
Supplement: Supplementary Fig. 3. — Changes in 3D chromatin organization can disturb gene expression. [file gi-23015-Supplementary-Figure-3.pdf]

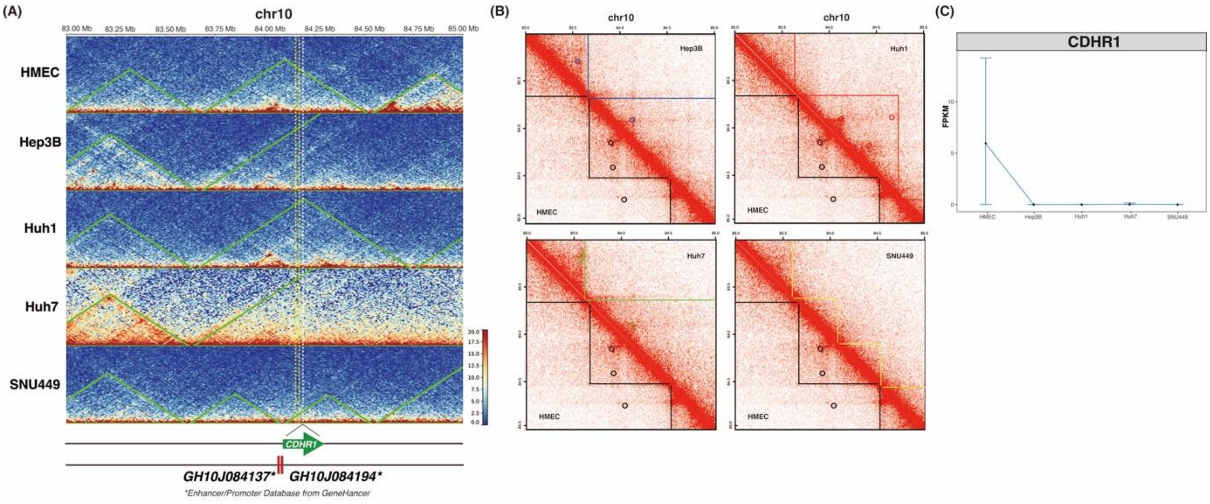

Supplementary Fig. 3. Changes in 3D chromatin organization can disturb gene expression. (A) Normalized Hi-C contact maps with topologically associated domains (TADs; green lines) indicated, spanning 83 to 85 Mb of chromosome 10 in the five cell lines. The white dashed line marks the *CDHR1* locus, and the yellow dashed lines mark the locations of enhancers. (B) Normalized Hi-C contact maps indicating TADs (straight lines) and chromatin loops (dots) of hepatocellular carcinoma cell (HCC) cell lines and human mammary epithelial cells (HMECs). The TAD boundaries of each cell line and chromatin loops are marked with the following colors: HMEC, black; Hep3B, blue; Huh1, red; Huh7, green; and SNU449, yellow. (C) FPKM (fragments per kilobase of transcript per million) gene-level plot of *CDHR1* in HMECs and HCC cell lines.
